# Health of Emigrant Ships

**Published:** 1854-08

**Authors:** 


### HEALTH OF EMIGRANT SHIPS.

This subject, so important to our great seaport towns and so interesting to humanity, has at last attracted the attention of Congress. An able and exceedingly valuable report was made to the Senate on the 2nd of this month, by Hon. HAMILTON FISH, of New York, embracing an analysis of a flood of facts collected by the committee appointed to investigate the matter. A frightful mortality, it is well known, has attended the poor who have come in such multitudes to our shores from the States of Europe during the last few years—a mortality as unnecessary as it is disgraceful to our age. It has resulted from the carelessness or cupidity of the owners of the emigrant ships—the want of ventilation, and of exercise in the open air on deck, overcrowding, insufficient or bad food, and the disgusting filth suffered to accumulate. Of 36,950 passengers in ships sailing from Europe in the last four months of the last year, 1,933 died of cholera before reaching New York, and 457 were sent to the hospital on landing, there probably to terminate their miserable existence, making nearly two per cent. of deaths among the whole number of persons who had embarked for the New World. The ships in which the pestilence broke out were those, as might have been predicted, which were most crowded with passengers, the amount of disease being in proportion to the crowding.

To provide the remedy for this shocking waste of human life, the committee recommended a number of stringent rules and regulations, which the captain has the power to enforce. Passenger ships are required to have space on the upper deck for a prominade for air and exercise; the number of passengers is to be limited; privies are to be increased in number, and those for females to be separated from those for male passengers. The captain has supervision over the culinary department, and is responsible for the cooking and distribution of the provisions, at stated hours. For failure in this duty he may be fined $1,000 and imprisoned for one year; and, in addition, each passenger may recover three dollars per day for each day of short allowance. But the most important and effective provision of the bill proposed, is that which makes it the pecuniary interest of the ship to bring its passengers safely to port, by compelling a forfeiture of the passage money of each one who dies on the voyage, of whatever disease. This is an admirable provision, which will not only insure attention to the comfort and health of the passengers, but will keep out the infirm and diseased, whose presence on board a crowded ship is calculated to breed infection. Such a law is in force in England and has been found to produce the happiest results in the British passenger trade. At one time, nearly one half of the criminal emigrants from England to Botany Bay died on the voyage; but a law was enacted, that passage money should be paid only for those passengers who were landed in safety, and the deaths declined, the first year, to one and a half per cent.

We trust that on the re-assembling of Congress, the bill submitted by Mr. FISH will be enacted. It is wise, humane, and important beyond any measure that, for many months, has come before our National Legislature. The operation of such a law cannot fail to be beneficent in the highest degree.

